# The relationships between inflammatory biomarkers, plaque characteristics, and macrophage clusters in coronary plaque: a quantitative assessment of macrophages based on optical coherence tomography

**DOI:** 10.3389/fcvm.2025.1625239

**Published:** 2025-06-25

**Authors:** Chunwei Liu, Fan Yang, Yuecheng Hu, Le Wang, Ximing Li, Hongliang Cong, Jingxia Zhang

**Affiliations:** ^1^ Department of Cardiology, Tianjin Chest Hospital, Tianjin University, Tianjin, China; ^2^ Department of Diagnostic Ultrasound, National Clinical Research Center of Cancer, Key Laboratory of Cancer Prevention and Therapy, Tianjin Medical University Cancer Institute and Hospital, Tianjin, China

**Keywords:** monocyte, high-density lipoprotein cholesterol, monocyte-to-HDL ratio, lipid index, macrophage cluster

## Abstract

**Background:**

Quantitative assessment of macrophage accumulation is appealing in evaluating plaque inflammation. In optical coherence tomography (OCT) imaging, local macrophage clusters may be a feasible marker for macrophage quantification.

**Methods:**

404 patients presenting with acute coronary syndrome who underwent OCT evaluation were included. This study aims to assess the relationships between systemic inflammatory biomarkers [including monocytes, high-density lipoprotein cholesterol (HDL-C), and monocyte-to-HDL ratio (MHR)], plaque characteristics, and local macrophage clusters in coronary plaque.

**Results:**

Macrophage clusters were present in 218 patients, with a median arc value of 72° (50°–163°). Patients with macrophage clusters showed markedly higher levels of inflammatory biomarkers and plaque vulnerability. Multivariate logistic regression analysis demonstrated that MHR, lipid index, and microchannel were independently associated with the presence of macrophage clusters. The DeLong test showed the area under the curve of the above three combined indicators was significantly larger than that of single indicators (0.774 vs. 0.692, 0.665, 0.624, respectively, *p* < 0.001). The macrophage cluster arc correlated positively with MHR and lipid index (*r* = 0.219, *p* = 0.001; and *r* = 0.229, *p* = 0.001, respectively). More superficial macrophage infiltration, thin cap fibroatheromas, plaque rupture, and thinner fibrous cap thickness were observed in the large macrophage cluster group (>72°) compared to the small macrophage cluster group (50°–72°). The macrophage cluster arc in the low MHR + lipid index group was significantly lower than that in the high MHR + lipid index group (68° ± 17° vs. 84° ± 26°, *p* = 0.001). Multiple linear regression analysis demonstrated that MHR, age, and lipid index were independently associated with macrophage cluster arc. In subgroup analysis stratified by clinical presentation and high-sensitivity C-reactive protein level, higher MHR and lipid index levels were observed in large macrophage clusters than in the non-macrophage cluster group, irrespective of the inflammation background.

**Conclusions:**

The macrophage cluster was a valuable index for quantifying local plaque inflammation. MHR, lipid index, and microchannel were independently associated with macrophage clusters. Large macrophage clusters were independently associated with high MHR and high lipid plaque burden.

## Graphical Abstract

Local macrophage clusters are independently associated with monocyte to high-density lipoprotein-cholesterol ratio, microchannel, and lipid plaque burden. 
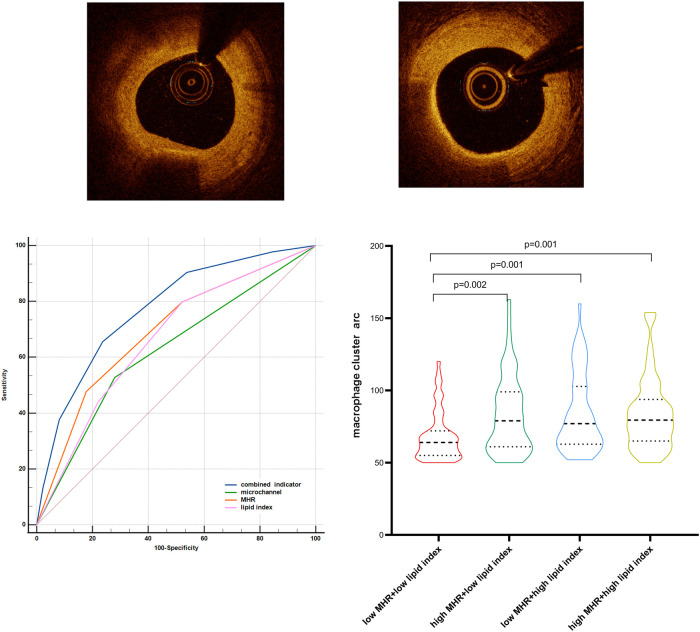

## Introduction

Macrophage-mediated inflammation plays an essential role in the pathogenesis of atherosclerosis and vulnerable plaque formation (1). The possibility of quantification of local plaque inflammation has always been appealing (2). Intravascular optical coherence tomography (OCT) enables high-resolution assessment of coronary plaque characteristics. Previous studies have shown that macrophages can be quantitatively measured by local OCT signal intensity (normalized standard deviation, NSD) originating from the reflectivity differences between the macrophages and the surrounding cap matrix. However, concerns have been raised about the influence of other plaque components that generate a high NSD, such as internal elastic lamina, cholesterol crystals, and small calcifications (3). Macrophage accumulation is prevalent in vulnerable plaques (4); however, as suggested by Tearney et al. (5), The isolated few bright spot signals of macrophages are probably not clinically significant; only a large accumulation of macrophage clusters may be of more concern. The concept of “macrophage clusters” has been attracting increasing attention in quantifying local macrophage accumulation (6). Recently, *post-hoc* analysis of the CLIMA study has shown that large macrophage clusters (>67°) in the non-culprit left anterior descending were associated with an increased risk of one-year MACE (7, 8). However, there is currently no research on macrophage clusters in the culprit lesions.

The relationship between local and systemic markers of inflammation has always been controversial. Previous small-scale OCT studies had reported that macrophage infiltration correlated with peripheral white blood count and C-reactive protein (CRP) in the culprit coronary plaque (9, 10). However, no significant correlation between macrophage clusters and CRP was observed in the *post-hoc* analysis of the CLIMA study (6). Macrophages derived from migrated monocytes engulfed large amounts of oxidized low-density lipoprotein cholesterol (LDL-C) to generate foamy cells and form lipid plaque (11). High-density lipoprotein cholesterol (HDL-C) exhibits antiatherogenic functions by inhibiting the inflammatory signaling of monocytes/macrophages and LDL oxidation (12). As a novel inflammation biomarker, monocyte-to-HDL ratio (MHR) has been a hallmark for predicting atherosclerosis development, progression, and cardiovascular outcomes (13, 14). In this current study, we aim to explore the application of macrophage clusters in the quantitative analysis of coronary plaque inflammation and evaluate the relationships between systemic inflammatory biomarkers (including monocytes, HDL-C, and MHR), plaque characteristics, and local macrophage clusters in acute coronary syndrome (ACS).

## Methods

### Study population

**Figure 1:**
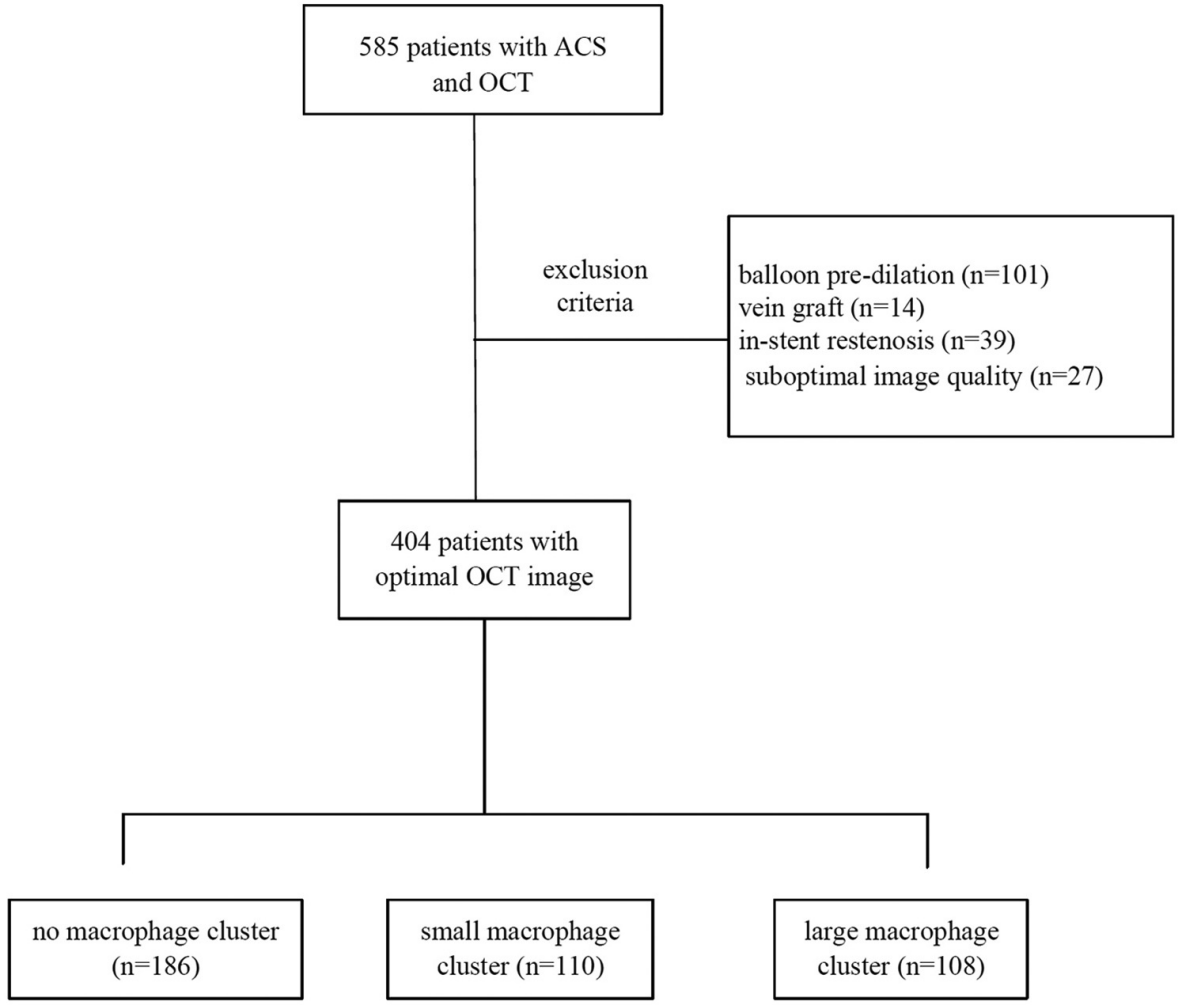
Study flow chart.

In this retrospective, single-center study, 585 patients presenting ACS who underwent an OCT examination during a clinically indicated coronary angiogram between Jan 2021 and Jan 2023 were identified in Tianjin Chest Hospital (Tianjin, China). Patients with OCT evaluation of the culprit vessel after balloon pre-dilation [*n* = 101, 48 for ST-segment elevation myocardial infarction (STEMI), 40 for non-ST-segment elevation myocardial infarction (NSTEMI), and 13 for unstable angina pectoris (UAP)], vein graft (*n* = 14), in-stent restenosis (*n* = 39), or suboptimal image quality (*n* = 27) were excluded. Finally, 404 patients were included in the present study (Figure 1). This retrospective study complied with the Declaration of Helsinki and was approved by the Ethics Committee of the Institute of Tianjin Chest Hospital. The data are anonymous, and the requirement for informed consent was therefore waived.

### OCT image acquisition and analysis

Coronary angiographies (CAG) and OCT examination were performed according to the standard techniques, and the culprit lesions were identified based on ECG and CAG findings. A commercially available OCT system (Dragonfly Duo or Dragonfly Optis, St Jude Medical, Inc., USA) was used for OCT examination. OCT images were analyzed based on available consensus documents by two blinded expert investigators independently (CWL and LW) using an off-line OCT console (St. Jude Medical). Disagreements were solved by consensus with a third investigator. OCT analysis was conducted both at the culprit lesion and the non-culprit lesion across the entire culprit vessel.

**Figure 2:**
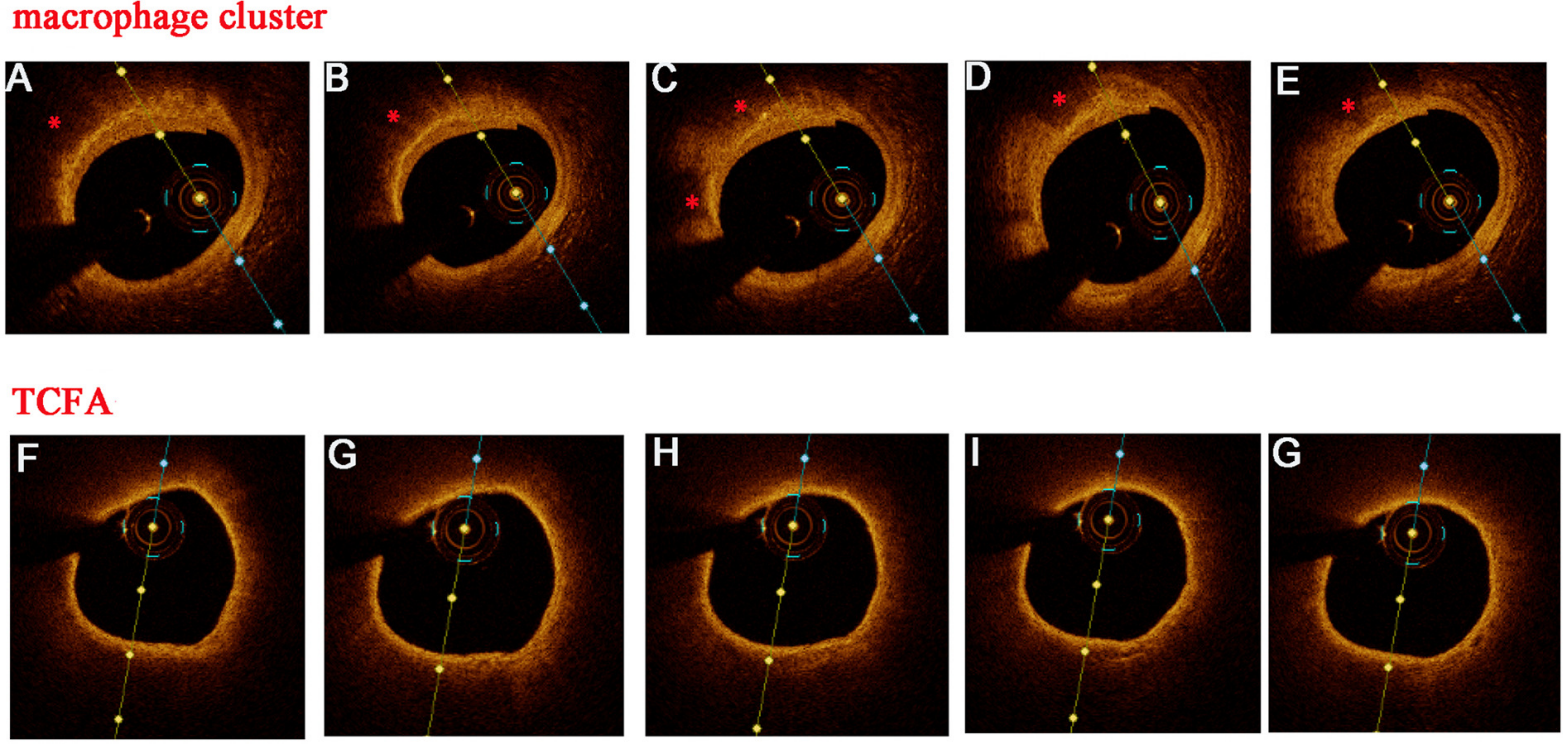
Discrimination between macrophage clusters (*) and TCFA in consecutive OCT images. Several OCT features supported superficial macrophage accumulation instead of TCFA: well-delineated, sharp radial borders, visualized underlying tissue in several adjacent frames, and a rapid change in appearance from frame to frame **(A–E**). In contrast, this were no sharp radial borders and rapid changes from frame to frame **(F–G)** in TCFA.

Lipid plaque was defined as a signal-poor region diffusely bordered by overlying signal-rich bands with a lipid arc of >180°. Thin cap fibroatheromas (TCFA) were described as a lipid-rich plaque with the thinnest fibrous cap ≤65 μm and lipid arc of >90° (15). The lipid index was defined as the mean lipid arc multiplied by the lipid length along the culprit vessel (culprit + non-culprit lesions). Macrophages were defined as signal-rich, distinct, or confluent punctate regions that exceed the intensity of background speckle noise (16) or strong linear OCT images on plaque surfaces accompanied by high attenuation (17). A macrophage cluster was defined as a large accumulation of confluent bright spots on plaque surfaces, accompanied by high attenuation (5). Superficial macrophage clusters may be misidentified as TCFA because of high attenuation (18). Figure 2 illustrates the distinguishing features between the macrophage clusters and TCFA in consecutive OCT images. The largest circumferential extension at the plaque cross-section was used for the analysis of multi-focal macrophage clusters (discrete, spatially separated aggregates of macrophages). The detailed information of the OCT examination is listed in the supplemental file.

### Inflammation and lipid biomarkers

Blood samples were collected before the OCT examination. The systemic inflammation and lipid biomarkers were retrieved from the medical records at our medical center, including white blood cells (WBC), neutrophils, lymphocytes, monocytes, platelets, mean platelet volume, hemoglobin, neutrophil/lymphocyte ratio (NLR), platelet/lymphocyte ratio (PLR), high-sensitivity C-reactive protein (hs-CRP), total cholesterol (TC), triglyceride (TG), LDL, HDL, MHR, neutrophil/HDL ratio (NHR) and LDL/HDL ratio.

### Subgroup analysis

The systemic inflammation and plaque vulnerability differed significantly between UAP, NSTEMI, and STEMI, so a subgroup analysis was conducted based on clinical presentation. Moreover, individuals were divided into two groups based on hs-CRP levels of <2 mg/L or ≥2 mg/L, and the inflammation biomarkers and plaque vulnerability were assessed at different hs-CRP levels. In addition, we divided patients with macrophage clusters into four groups based on the median value of MHR and lipid index.

### Statistical analysis

The Kolmogorov–Smirnov test assessed variable distribution. Continuous variables are expressed as mean ± SD or median (interquartile range, 25%–75%), and categorical variables are presented as frequencies and percentages. An independent samples t-test, Mann–Whitney U test, or one-way analysis of variance was used to compare continuous variables. In contrast, the discrete values were compared using the Chi-square test or Fisher's exact test. The receiver operating characteristic curve (ROC) analysis evaluates the predictive significance of inflammation and lipid biomarkers for the presence of macrophage clusters. Intra-observer reliability was assessed using the Cohen Kappa test for categorical variables and the intraclass correlation test for continuous variables. The associations among inflammation and lipid biomarkers, plaque vulnerability, and macrophage clusters were analyzed using a logistic regression model with a stepwise selection of the variables exhibiting *p* < 0.05, no obvious collinearity (Spearman's *r* < 0.7 and variance inflation factor <5), and overfitting in the univariate analysis. Multiple linear regression analyses were performed to assess the risk factors for macrophage cluster arcs in patients with macrophage clusters. Spearman's correlation coefficients analyzed the relationship between inflammation biomarkers and macrophage clusters. A Fisher's Z transformation was performed to test for differences between correlations. A two-tailed *p*-value of <0.05 was considered statistically significant. All data were analyzed using SPSS Statistics (IBM, version 26), Medcalc (MedCalc Software Ltd, version 23), and GraphPad Prism (GraphPad Software Ltd, version 8).

## Results

### Baseline characteristics of macrophage accumulation

**Figure 3:**
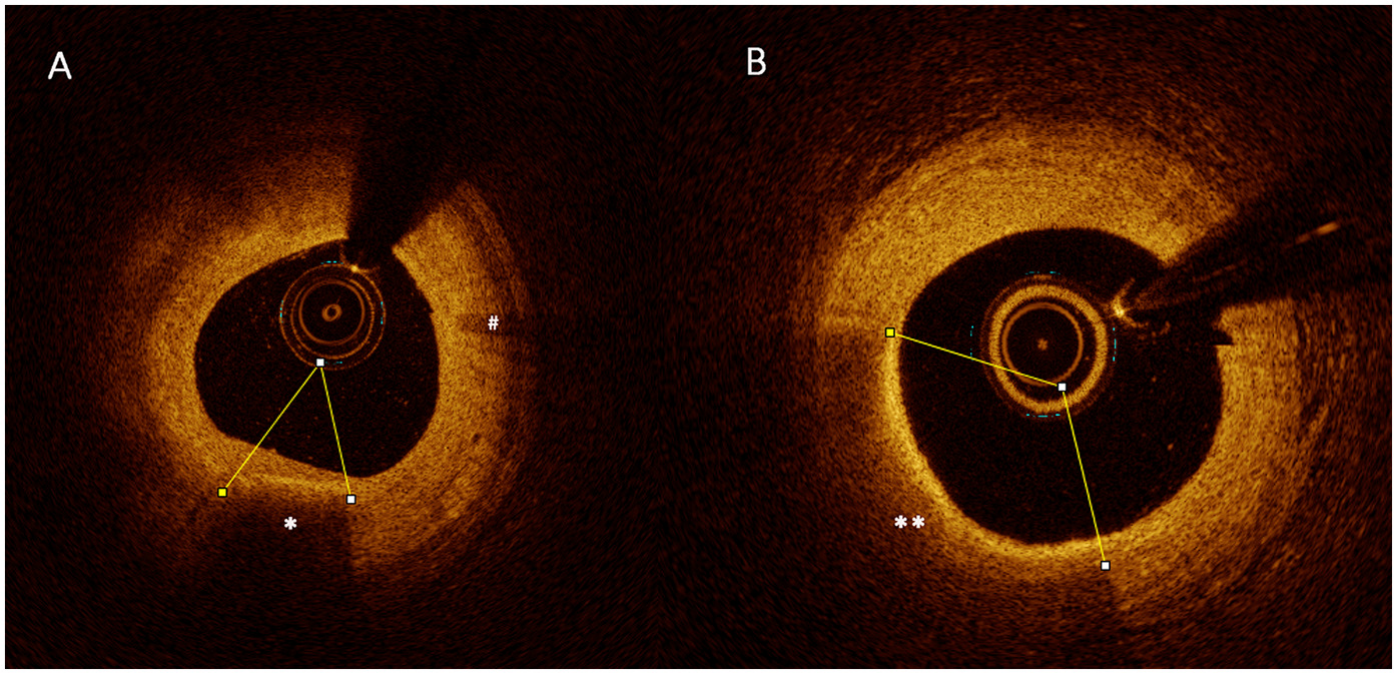
OCT example of large macrophage clusters (**) **(B)**, small macrophage clusters (*) **(A)**, and a few isolated bright spot signals of macrophage infiltration (**^#^**).

A total of 404 patients with ACS were included in the present study. OCT detected small bright spot signals of macrophage infiltration in 378 patients (94%). A large accumulation of confluent bright spots (macrophage cluster) was present in 218 patients (54%), with a median circumferential extension value of 72° (range: 50°–163°, Figure 3). The presence of macrophage clusters was defined as circumferential extension > 50° based on our dataset, and the patients were divided into three groups according to the median value of circumferential extension: no macrophage cluster (<50°), small macrophage cluster (50°–72°) and large macrophage cluster (>72°). The minimum distance from macrophage accumulation to the lumen was shorter in large macrophage clusters compared with small macrophage clusters (94.5 ± 31.5 vs. 143.5 ± 54.2 μm, *p* < 0.001). Multi-focal macrophage clusters were identified in 127 patients, and the number of macrophage clusters per vessel was significantly higher in patients with large macrophage clusters than in patients with small macrophage clusters (2.7 ± 1.2 vs. 1.4 ± 0.6, *p* < 0.001). Since there was low accuracy in the culprit lesion with a large necrotic core or massive thrombus, 71% of macrophage clusters were identified within fibroatheroma in the non-culprit lesion in this present study. The circumferential extension did not significantly differ between culprit and non-culprit lesions (*p* > 0.05). The minimum distance from macrophage accumulation to the lumen was smaller in culprit lesions compared with the non-culprit lesion (84.8 ± 17.7 vs. 129.8 ± 50.0 μm, *p* = 0.005). There were good inter-observer agreements for macrophage assessment, with R of 0.96 for circumferential extension and R of 0.94 for minimum distance.

### Macrophage clusters vs. no macrophage clusters

The comparison between baseline patient characteristics and laboratory data in patients with macrophage clusters vs. those without macrophage clusters is shown in Table 1. Patients with macrophage clusters were much younger, more male, and more frequently of acute myocardial infarction (AMI) and multivessel disease. Meanwhile, laboratory ﬁndings showed markedly higher levels of systemic inflammation, including WBC, neutrophils, lymphocytes, monocytes, hemoglobin, NHR, MHR, hs-CRP, and LDL/HDL, and lower levels of HDL-C and left ventricular ejection fraction (LVEF) (*p* < 0.05).

**Table 1:** Comparison of clinical characteristics, systemic inflammation biomarkers between macrophage cluster groups.

Variable	All patients (*n* = 404)	No MØC (*n* = 186)	MØC (*n* = 218)	*P*_with vs. without_	Small MØC (*n* = 110)	Large MØC (*n* = 108)	*P*_small vs. large_
Male	301	124 (67%)	177 (81%)	0.001*	84 (76%)	93 (86%)	0.066
Age	61.7 ± 10.8	62.8 ± 10.4	60.7 ± 11.1	0.049*	61.7 ± 10.0	59.7 ± 12.1	0.180
Diabetes mellitus	123	57 (31%)	66 (30%)	0.936	36 (33%)	30 (28%)	0.426
hypertension	247	114 (61%)	133 (61%)	0.954	71 (65%)	62 (57%)	0.280
current smoker	186	82 (44%)	117 (54%)	0.055	54 (49%)	63 (58%)	0.171
Clinical diagnosis							
UAP	284	147 (79%)	137 (63%)	0.002*	75 (68%)	62 (58%)	0.254
STEMI	63	20 (11%)	43 (20%)		19 (17%)	24 (22%)	
NSTEMI	57	19 (10%)	38 (17%)		16 (15%)	22 (20%)	
Prior PCI	57	28 (15%)	29 (13%)	0.614	21 (19%)	8 (7%)	0.011*
Prior AMI	37	17 (9%)	20 (9%)	0.990	13 (12%)	7 (7%)	0.172
WBC (10^9^/L)	7.15 ± 2.21	6.60 ± 1.93	7.61 ± 2.32	<0.001*	7.20 ± 1.91	8.03 ± 2.62	0.008*
Neutrophils	4.71 ± 1.99	4.35 ± 1.82	5.02 ± 2.08	0.001*	4.64 ± 1.80	5.40 ± 2.28	0.007*
Lymphocytes	1.82 ± 0.62	1.71 ± 0.61	1.90 ± 0.62	0.002*	1.87 ± 0.54	1.93 ± 0.68	0.481
Monocytes	0.443 ± 0.166	0.383 ± 0.131	0.495 ± 0.175	<0.001*	0.464 ± 0.150	0.526 ± 0.193	0.010*
Platelets (10^9^/L)	226 ± 63	225 ± 65	226 ± 62	0.935	222 ± 68	230 ± 56	0.323
MPV (fl)	10.1 ± 0.8	10.1 ± 0.8	10.2 ± 0.9	0.104	10.2 ± 1.0	10.3 ± 0.9	0.787
Hemoglobin (g/L)	139 ± 15	137 ± 15	141 ± 14	0.035*	140 ± 14	141 ± 15	0.426
NLR	3.06 ± 2.67	3.10 ± 3.04	3.02 ± 2.31	0.772	2.91 ± 2.78	3.14 ± 1.71	0.469
PLR	140 ± 76	150 ± 89	132 ± 63	0.018*	129 ± 56	135 ± 70	0.505
hs-CRP (mg/l)	1.27 (0.60–3.84)	1.12 (0.54–2.89)	1.56 (0.62–5.43)	0.004*	1.16 (0.56–3.44)	2.70 (0.87–7.13)	0.005*
FBG (mmol/L)	6.12 ± 2.04	6.00 ± 2.06	6.22 ± 2.02	0.289	6.33 ± 2.11	6.10 ± 1.93	0.386
TC (mmol/L)	4.09 ± 1.05	4.01 ± 1.07	4.17 ± 1.02	0.138	4.25 ± 1.07	4.08 ± 0.98	0.233
TG (mmol/lL)	1.50 (1.10–2.17)	1.43 (1.06–2.04)	1.58 (1.14–2.25)	0.060	1.54 (1.12–2.17)	1.63 (1.16–2.30)	0.285
LDLC (mmol/L)	2.58 ± 0.92	2.49 ± 0.94	2.67 ± 0.90	0.057	2.72 ± 0.91	2.61 ± 0.89	0.353
HDLC (mmol/L)	1.03 ± 0.23	1.07 ± 0.23	1.00 ± 0.22	0.002*	1.04 ± 0.21	0.95 ± 0.21	0.003*
NHR	4.82 ± 2.34	4.25 ± 1.93	5.30 ± 2.54	<0.001*	4.69 ± 2.06	5.93 ± 2.83	<0.001*
MHR	0.445 ± 0.207	0.374 ± 0.150	0.524 ± 0.223	<0.001*	0.471 ± 0.191	0.578 ± 0.241	<0.001*
LDL/HDL	2.62 ± 1.04	2.42 ± 0.97	2.78 ± 1.06	<0.001*	2.72 ± 1.04	2.85 ± 1.08	0.385
eGFR (ml/min1.73 m^2^)	88.6 ± 14.9	88.5 ± 15.5	88.7 ± 14.5	0.867	87.0 ± 14.2	90.5 ± 14.7	0.080
Uric acid (umol/L)	341 ± 172	337 ± 237	345 ± 86	0.609	345 ± 88	346 ± 84	0.957
D-dimer (μg/ml)	0.23 (0.19–0.32)	0.23 (0.19–0.31)	0.24 (0.19–0.37)	0.261	0.22 (0.19–0.37)	0.25 (0.19–0.37)	0.395
LVEF (%)	58.3 ± 6.2	59.1 ± 5.1	57.7 ± 6.9	0.017*	57.7 ± 6.8	57.6 ± 7.0	0.940
Medications							
Statins	133	65 (35%)	68 (30%)	0.267	36 (33%)	32 (30%)	0.622
β-blockers	142	71 (38%)	69 (32%)	0.170	34 (34%)	35 (32%)	0.807
ACEI/ARB	110	51 (27%)	59 (27%)	0.936	31 (28%)	28 (26%)	0.708
CCB	127	66 (36%)	61 (28%)	0.105	33 (30%)	29 (27%)	0.606
Aspirin	346	161 (87%)	185 (85%)	0.628	91 (83%)	94 (87%)	0.375

Values are mean ± SD, *n* (%), or median (25th–75th percentile).

MØC, macrophage cluster; UAP, unstable angina pectoris; NSTEMI, non-ST-segment elevation myocardial infarction; STEMI, ST-segment elevation myocardial infarction; PCI, percutaneous coronary intervention; WBC, white blood cells; MPV, mean platelet volume; NLR, neutrophil/lymphocyte ratio; PLR, platelet/lymphocyte ratio; hs-CRP, high-sensitivity C-reactive protein; FBG, fasting blood glucose; TC, total cholesterol; TG, triglyceride; LDLC, low-density lipoprotein cholesterol; HDLC, high-density lipoprotein cholesterol; NHR, neutrophil/HDL ratio; MHR, monocyte/HDL ratio; eGFR, estimated glomerular filtration rate; BNP, brain natriuretic peptide; LVEF, left ventricular ejection fraction; ACEI, angiotensin-converting enzyme inhibitors; ARB, angiotensin receptor blocker; CCB, calcium channel blockers.

* *P* < 0.05.

Table 2 summarizes the OCT analysis of culprit vessels. Patients with macrophage clusters showed a larger lipid index, thinner fibrous cap thickness (FCT), more plaque rupture, layered plaque, TCFA, cholesterol crystals, and microchannels compared to those without macrophage clusters, but no differences in thrombus, minimal lumen area, calcification index, calcification thickness, and calcification types.

**Table 2:** Comparison of angiographic characteristics and OCT findings between macrophage cluster groups.

Variable	All patients (*n* = 404)	No MØC (*n* = 186)	MØC (*n* = 218)	*P*_with vs. without_	Small MØC (*n* = 110)	large MØC (*n* = 108)	*P*_small vs. large_
Culprit vessel							
LAD	282	144 (77%)	138 (63%)	0.013*	73 (66%)	65 (60%)	0.296
RCA	88	33 (18%)	55 (25%)		26 (24%)	29 (27%)	
LCX	31	8 (4%)	23 (11%)		9 (8%)	14 (13%)	
LM	3	1 (1%)	2 (1%)		2 (2%)	0 (0%)	
Multivessel disease							
One vessel	119	71 (38%)	48 (22%)	0.001*	25 (23%)	23 (21%)	0.442
Two vessels	124	56 (30%)	68 (31%)		38 (34%)	30 (28%)	
Three vessels	161	59 (32%)	102 (47%)		47 (43%)	55 (51%)	
OCT findings							
Plaque rupture	72	21 (11%)	51 (23%)	0.002*	19 (17%)	32 (30%)	0.031*
Plaque erosion	12	7 (4%)	5 (2%)	0.386	4 (4%)	1 (1%)	0.181
TCFA	100	31 (17%)	69 (32%)	0.001*	24 (22%)	45 (42%)	0.002*
Thrombus	75				44		
Red thrombus	22	10 (5%)	12 (6%)	0.254	4 (4%)	8 (7%)	0.099
White thrombus	48	17 (9%)	31 (14%)		18 (16%)	13 (12%)	
Mix thrombus	5	1 (1%)	4 (2%)		0 (0%)	4 (4%)	
MLA	2.11 ± 1.07	2.22 ± 1.23	2.02 ± 0.90	0.053	2.04 ± 0.81	1.99 ± 0.98	0.668
Lipid length	35.7 ± 14.7	30.6 ± 14.1	40.0 ± 13.7	<0.001*	37.4 ± 13.4	42.6 ± 13.5	0.005*
Max lipid arc°	298 ± 64	284 ± 68	311 ± 59	<0.001*	298 ± 65	323 ± 49	0.002*
Lipid index	7,402 ± 3,433	6,170 ± 3,153	8,454 ± 3,318	<0.001*	7,732 ± 3,237	9,190 ± 3,251	0.001*
FCT (μm)	129 ± 76	141 ± 79	119 ± 73	0.006*	137 ± 81	102 ± 59	<0.001*
Layered plaque	130	48 (26%)	82 (38%)	0.011*	42 (38%)	40 (37%)	0.862
Cholesterol crystal	91	31 (17%)	60 (28%)	0.009*	28 (26%)	32 (30%)	0.490
Microchannel	167	52 (28%)	115 (53%)	<0.001*	55 (50%)	60 (56%)	0.411
Calcification length	7.0 (1.7–16.0)	7.5 (1.0–18.0)	6.8 (2.0–15.2)	0.724	7 (3.2–15.6)	6.4 (1.0–14.6)	0.327
Max calcification arc°	78 (35–118)	80 (31–133)	76 (44–109)	0.632	77 (52–104)	76 (26–110)	0.398
Max Calcification thickness (μm)	692 ± 448	729 ± 492	660 ± 397	0.130	696 ± 379	622 ± 415	0.172
Calcification index	425 (62–1,092)	516 (38–1,272)	391 (84–998)	0.458	393 (133–966)	378 (25–1,010)	0.364
Calcification types							
No calcification	77	40 (21%)	37 (17%)	0.624	13 (12%)	24 (22%)	0.059
Calcified protrusion	22	11 (6%)	11 (5%)		6 (5%)	5 (5%)	
Eruptive calcified nodules	27	11 (6%)	16 (7%)		12 (11%)	4 (4%)	
Superficial calcific sheet	278	124 (67%)	154 (71%)		79 (72%)	75 (69%)	

Values are mean ± SD, *n* (%), or median (25th–75th percentile).

MØC, macrophage cluster; LAD, left anterior descending; RCA, right coronary artery; LCX, left circumflex artery; LM, left main; TCFA, Thin cap fibroatheromas; MLA, minimal lumen area; FCT, fibrous cap thickness.

* *P* < 0.05.

A multivariate logistic regression analysis demonstrated that MHR [odds ratio (OR): 121.714; 95% CI: 14.939–991.673, *p* < 0.001], lipid index (OR: 3.381; 95% CI: 1.941–5.891, *p* < 0.001), and microchannel (OR: 2.634; 95% CI: 1.549–4.479, *p* < 0.001) were associated with the presence of macrophage clusters independently of age, sex, clinical presentation, multivessel disease, NHR, PLR, hemoglobin, hs-CRP, LVEF, FCT, TCFA, plaque rupture, layered plaque and cholesterol crystal (Table 3).

**Table 3:** Multivariable logistic regression analysis for macrophage clusters.

Parameter	*P*	Odds ratio	95% CI
MHR	<0.001	121.714	14.939–991.673
Ln (Lipid index)	<0.001	3.381	1.941–5.891
Microchannel	<0.001	2.634	1.549–4.479
TCFA	0.070	1.941	0.948–3.975
NHR	0.219	0.900	0.761–1.065
PLR	0.154	0.997	0.993–1.001
age	0.656	0.994	0.969–1.020
male	0.778	0.913	0.484–1.721
UAP/NSTEMI/STEMI	0.459	1.161	0.782–1.725
multivessel disease	0.291	1.180	0.868–1.606
hemoglobin	0.871	1.002	0.982–1.021
hs-CRP	0.357	1.028	0.969–1.091
LVEF	0.431	0.982	0.938–1.028
Plaque rupture	0.936	0.970	0.459–2.049
Layered plaque	0.328	1.302	0.767–2.212
Cholesterol crystal	0.679	0.881	0.484–1.605
FCT	0.779	1.001	0.997–1.005

MHR, monocyte/HDL ratio; TCFA, Thin cap fibroatheromas; NHR, neutrophil/HDL ratio; PLR, platelet/lymphocyte ratio; UAP, unstable angina pectoris; NSTEMI, non-ST-segment elevation myocardial infarction; STEMI, ST-segment elevation myocardial infarction; hs-CRP, high-sensitivity C-reactive protein; LVEF, left ventricular ejection fraction; FCT, fibrous cap thickness.

**Figure 4:**
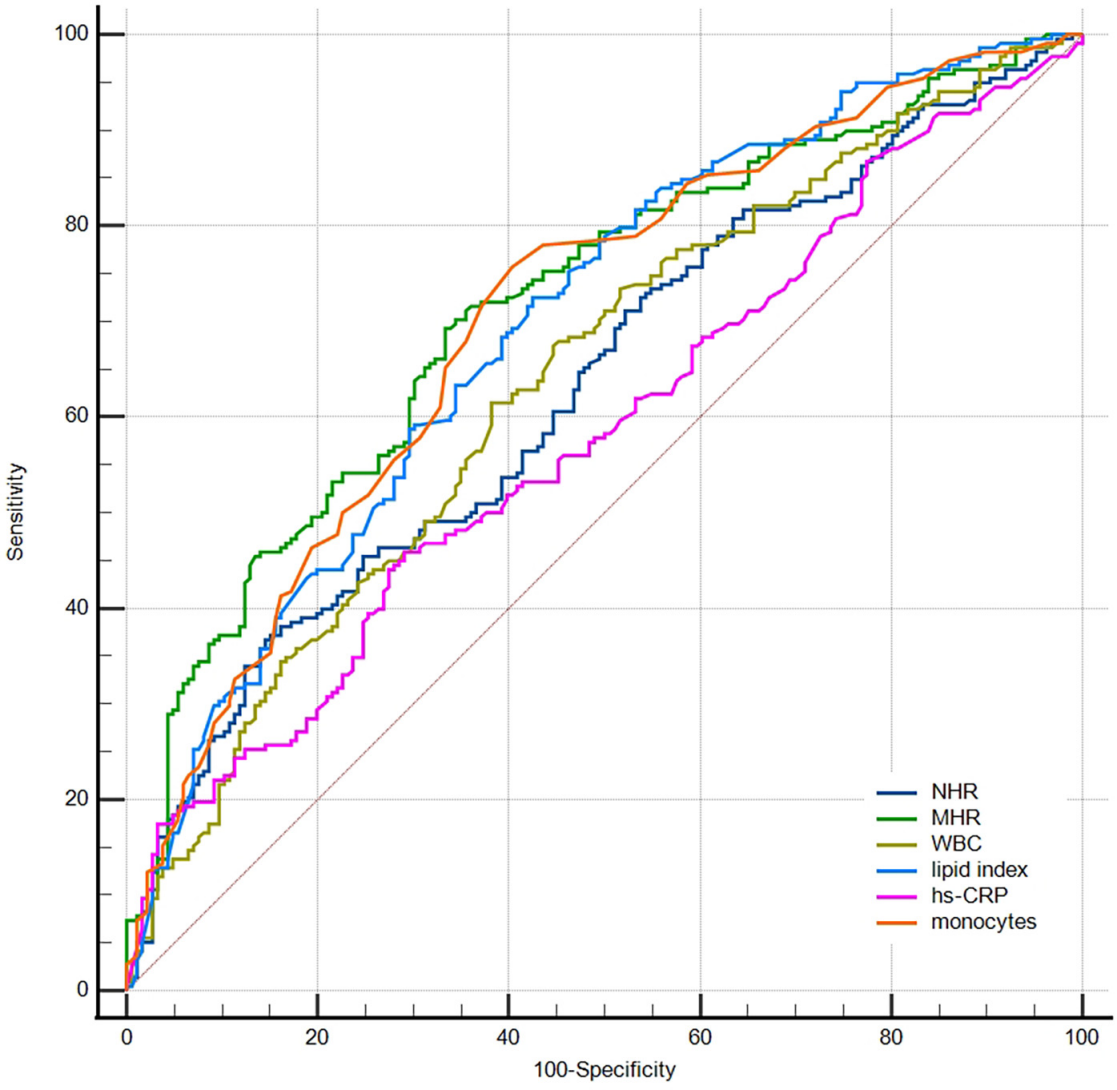
Receiver operator curves for prediction of macrophage clusters. NHR, neutrophil/HDL ratio; MHR, monocyte/HDL ratio; WBC, white blood cells; hs-CRP, high-sensitivity C-reactive protein.

ROC curves were produced to compare the predictive ability among inflammation biomarkers (Figure 4). The areas under the ROC curve (AUC) for MHR, monocytes, NHR, WBC, and hs-CRP were 0.72, 0.70, 0.63, 0.64, and 0.58, respectively. The DeLong test indicated that MHR and monocytes showed a significantly greater AUC than NHR, WBC, and hs-CRP (*p* < 0.001). The MHR level of 0.404 was the best cut-off point to predict macrophage clusters, with a sensitivity of 69% and specificity of 67%.

**Figure 5:**
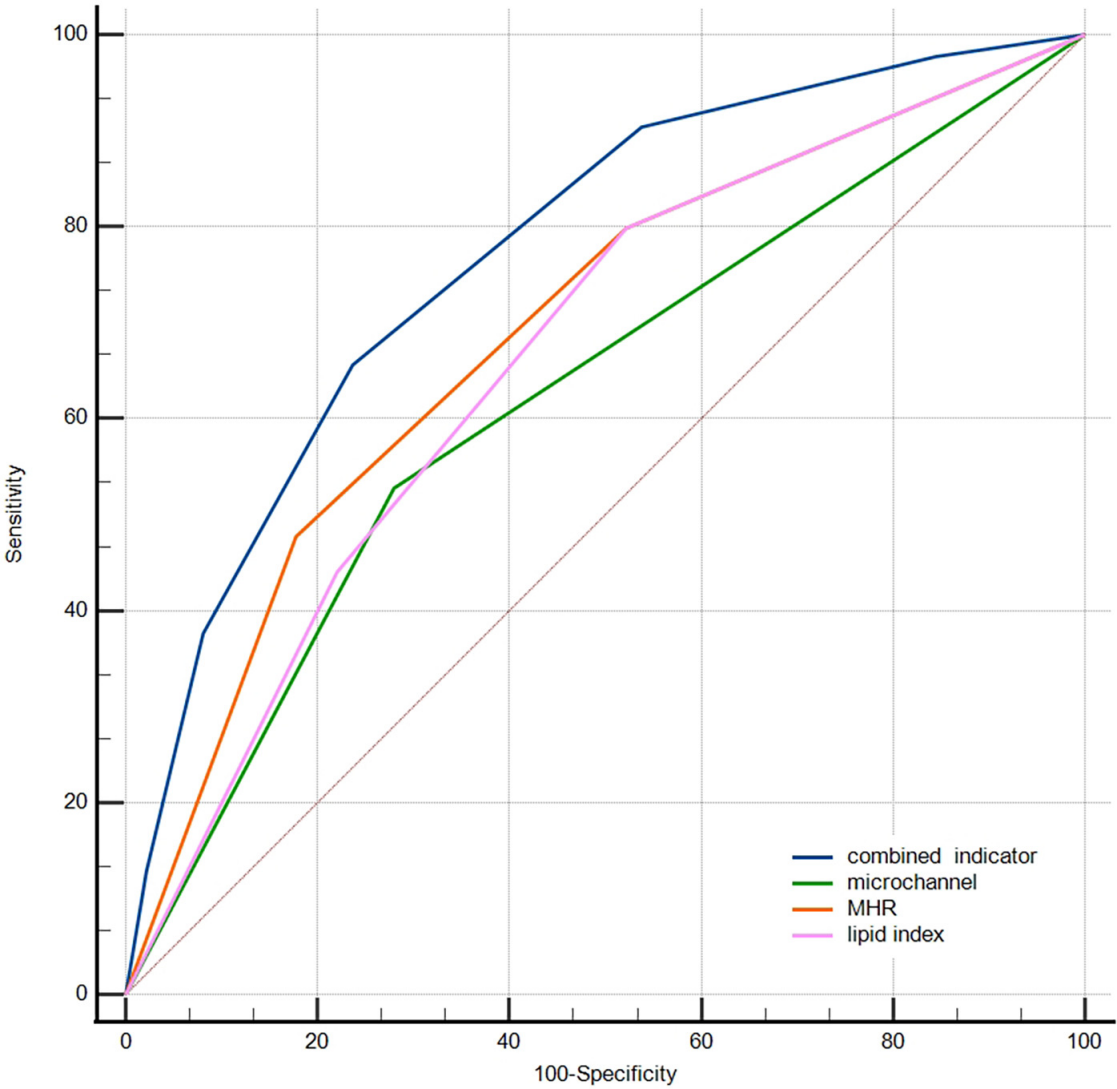
Comparison of the predictive abilities of MHR, lipid index, microchannels, and combined indicator for macrophage clusters. MHR, monocyte/HDL ratio.

The ROC curve was further used to compare the predictive abilities of microchannels (absence = 0, presence = 1 point), MHR tertiles, lipid index tertiles (low, medium, and high scores were 1, 2, and 3, respectively), and the combined three indicators for macrophage clusters (Figure 5). The DeLong test showed that the area under the curve of the combined indicator was significantly larger than that of the single indicators of MHR, lipid index, and microchannels (0.774 vs. 0.692, 0.665, 0.624, respectively, *p* < 0.001).

Moreover, MHR levels were positively correlated with hs-CRP (*r* = 0.384, *p* < 0.001) and TG (*r* = 0.222, *p* < 0.001). The levels of uric acid, lipid index, FCT, and calcification index were slightly correlated with the MHR level (*r* = 0.139, *p* = 0.005; *r* = 0.114, *p* = 0.022; *r* = −0.120, *p* = 0.016; *r* = −0.134, *p* = 0.007, respectively). A higher level of MHR was observed in plaque rupture (0.536 ± 0.257 vs. 0.438 ± 0.190, *p* = 0.003) but not in plaque erosion, TCFA, cholesterol crystals, and microchannels. Of note, the level of monocytes was not correlated with lipid index (*r* = 0.078, *p* = 0.117).

### Small vs. large macrophage clusters

Patients with large macrophage clusters had a higher prevalence of prior percutaneous coronary intervention (PCI) history, TCFA, and plaque rupture, higher levels of WBC, neutrophils, monocytes, MHR, NHR, hs-CRP, and lipid index, and thinner FCT than patients with small macrophage clusters (Table 2, *p* < 0.05). No significant differences were observed in layered plaque, plaque erosion, microchannel, and cholesterol crystal among the two groups. Patients with small macrophage clusters had a higher prevalence of calcified plaque (88% vs. 78%, *p* = 0.041). However, the calcified index and the proportions of calcified protrusion, eruptive calcified nodules, and superficial calcific sheet were comparable between the two groups. A statistically significant negative relationship existed between HDL-C and the macrophage cluster circumferential extension. In addition, neither TC, LDL-C, nor TG showed any significant relationship with the macrophage cluster circumferential extension.

Spearman's correlation coefficients demonstrated that the circumferential extension of the macrophage cluster had a positive correlation with MHR (*r* = 0.219, *p*= 0.001, *n* = 218), monocytes (*r* = 0.139, *p* = 0.041, *n* = 218), and lipid index (*r* = 0.229, *p* = 0.001, *n* = 218), and a negative correlation with HDL-C (*r* = −0.257, *p*< 0.001, *n* = 218). Using Fisher's z transformation for testing, the correlation between macrophage cluster arc and MHR was significantly higher than that of monocytes (*z* = 2.24, *p* = 0.025). Moreover, multiple linear regression analysis in patients with macrophage clusters demonstrated that MHR (standardized beta coefficient = 0.204, *p* = 0.002), age (standardized beta coefficients = −0.153, *p* = 0.021), and lipid index (standardized beta coefficients = 0.135, *p* = 0.043) were independently associated with the circumferential extension of the macrophage cluster (*F* = 7.194, *p* < 0.001, variance inflation factor <2, Table 4).

**Table 4:** Univariable and multiple linear regression analysis for factors related to macrophage cluster arc.

Parameter	Univariable model				Multivariable model			
	Nonstandardized	Standard	Standardized	*P*	Nonstandardized	Standard	Standardized	*P*
	Beta coefficient	Error	Beta coefficient		Beta coefficient	Error	Beta coefficient	
MHR	29.559	7.409	3.989	<0.001	22.976	7.348	0.204	0.002
age	−0.490	0.151	−0.216	0.001	−0.347	0.150	−0.153	0.021
Lipid index	0.002	0.001	0.199	0.003	0.001	0.001	0.135	0.043
FCT	−0.081	0.023	−0.235	<0.001	−0.040	0.028	−0.116	0.161
TCFA	12.465	3.581	0.230	0.001	5.361	4.567	0.099	0.242
Plaque rupture	12.749	3.949	0.215	0.001	1.842	4.419	0.031	0.677
hs-CRP	0.430	0.283	0.103	0.130				
MLA	1.464	1.913	0.052	0.445				
microchannel	1.707	3.427	0.034	0.619				

MHR, monocyte/HDL ratio; FCT, fibrous cap thickness; TCFA, thin cap fibroatheromas; hs-CRP, high-sensitivity C-reactive protein; MLA, minimal lumen area.

**Figure 6:**
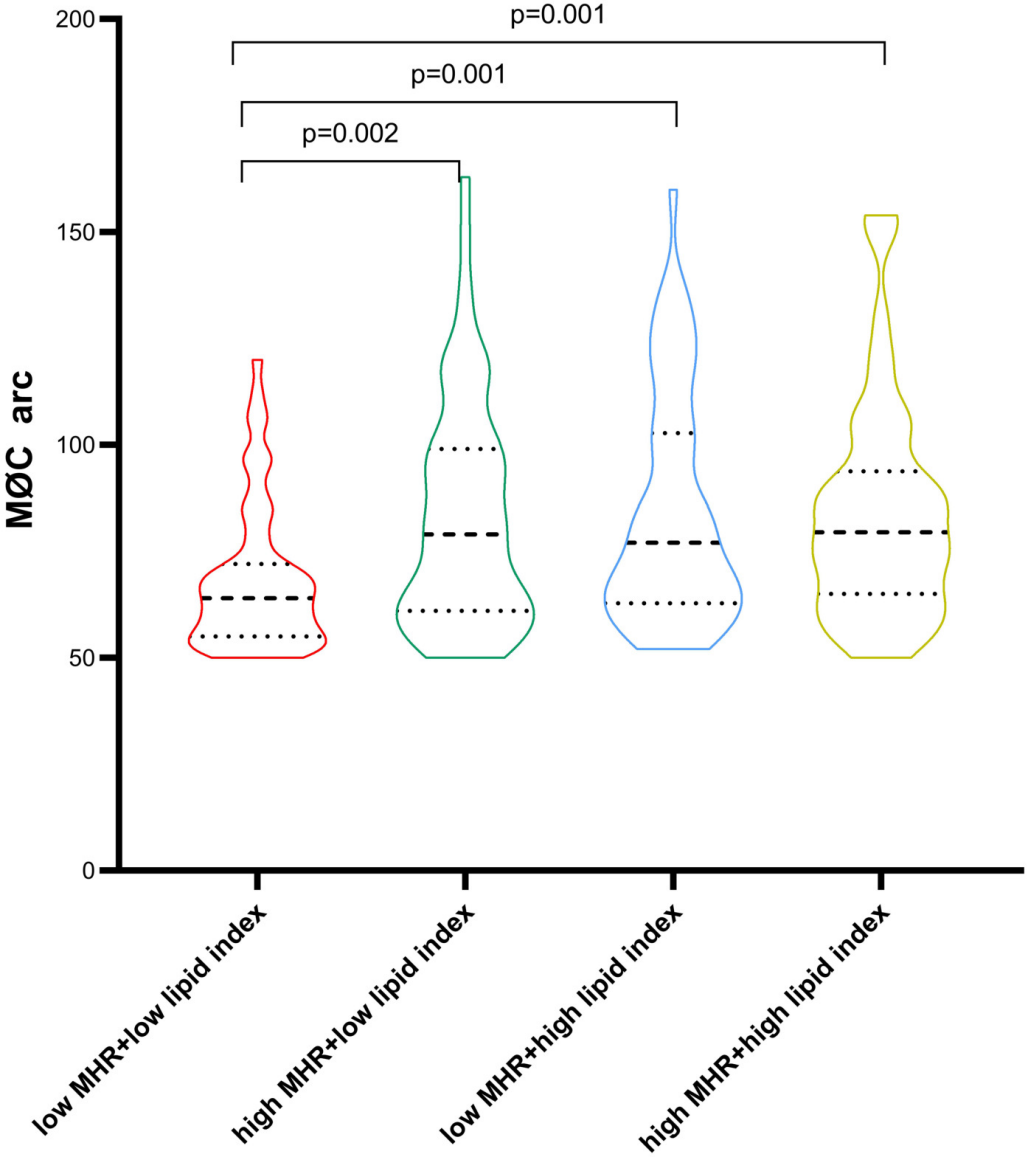
Comparison of macrophage cluster arc between groups stratified by the MHR and lipid index median value. MHR, monocyte/HDL ratio.

Patients with macrophage clusters were divided into four groups stratified by the median value of MHR and lipid index (0.484 and 8,262, respectively). The circumferential extension of macrophage clusters in the low MHR + low lipid index group was significantly lower than that in the low MHR + high lipid index, high MHR + low lipid index, and high MHR + high lipid index groups (68 ± 17, 83 ± 27, 84 ± 27, 84 ± 26, respectively, *p* = 0.001, Figure 6). Moreover, patients with high MHR + high lipid index had a larger number of macrophage clusters along the culprit's vessel than patients with the low MHR + low lipid index (2.2 ± 1.4 vs. 1.6 ± 0.9, *p* = 0.006). Of note, compared to the low MHR + low lipid index group, the high MHR + high lipid index group had a higher prevalence of plaque rupture (40% vs. 15%, *p* = 0.001), and thinner FCT (91.9 ± 45.2 vs. 135.4 ± 78.8 μm, *p* = 0.002). However, no significant differences were observed in TCFA, plaque erosion, layered plaque, microchannel, and cholesterol crystal among the four groups (Supplement Table S1). Moreover, the comparison of MHR, lipid index, and microchannel showed significant differences between groups based on the circumferential extension tertiles (64° and 86°, Supplement Table S2, *p* < 0.05).

### Subgroup analysis

**Figure 7:**
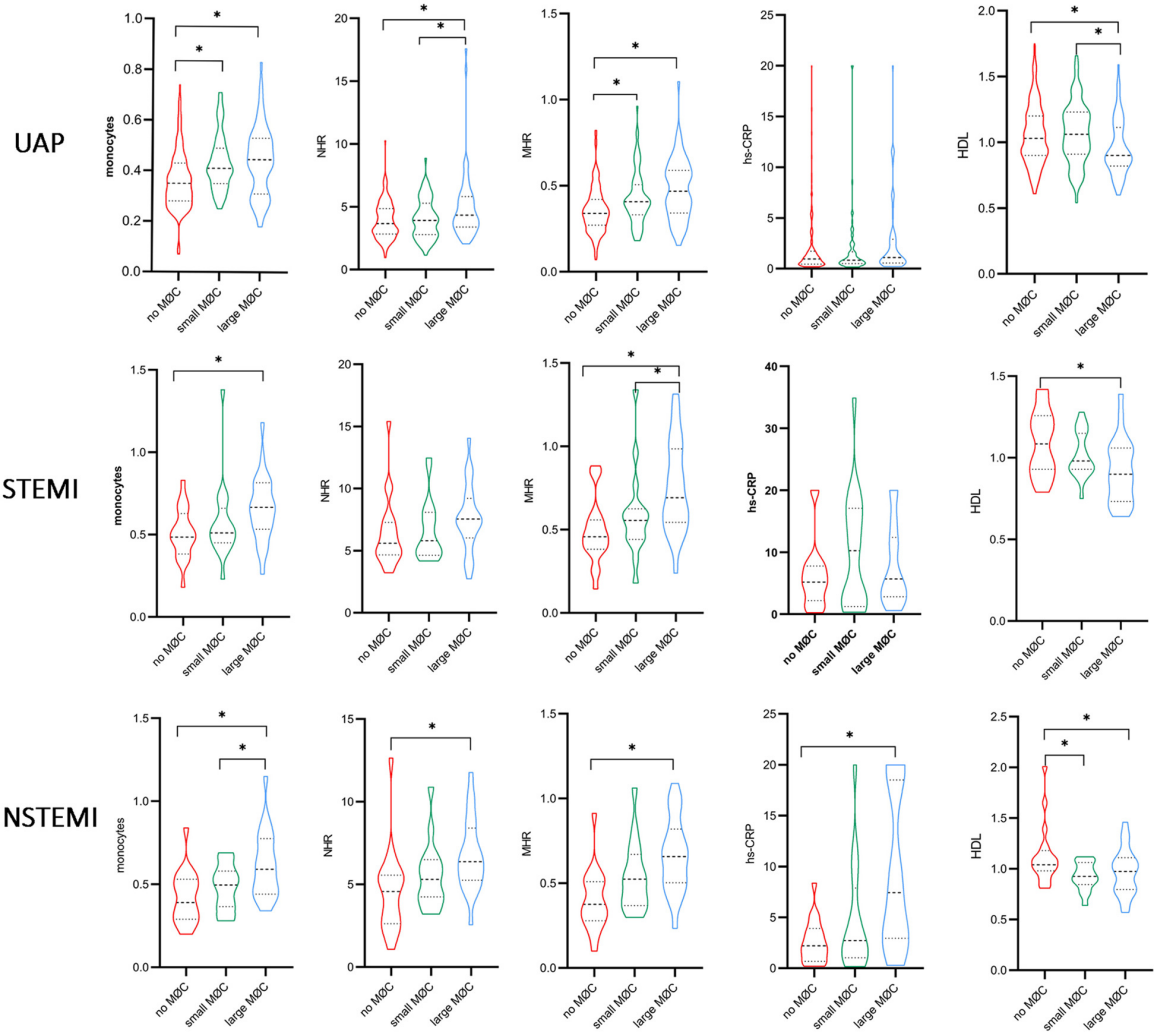
Comparison of inflammation biomarkers and plaque characteristics between macrophage cluster groups in patients with different clinical presentations. *indicated *p* < 0.05.

Notably, systemic inflammation levels (WBC, neutrophils, monocytes, NHR, MHR, and hs-CRP) showed a significant gradient from UAP to NSTEMI to STEMI (Supplement Table S3, *p* < 0.05). As illustrated in Figure 7, higher levels of monocytes, MHR, and lipid index and lower levels of HDL were observed in the large macrophage cluster group compared to the no macrophage cluster group, irrespective of clinical presentation. NHR, TCFA, and FCT exhibited significant differences between the large and no macrophage cluster groups in patients presenting with UAP and NSTEMI. In contrast, it was not significantly different in STEMI patients.

**Figure:**
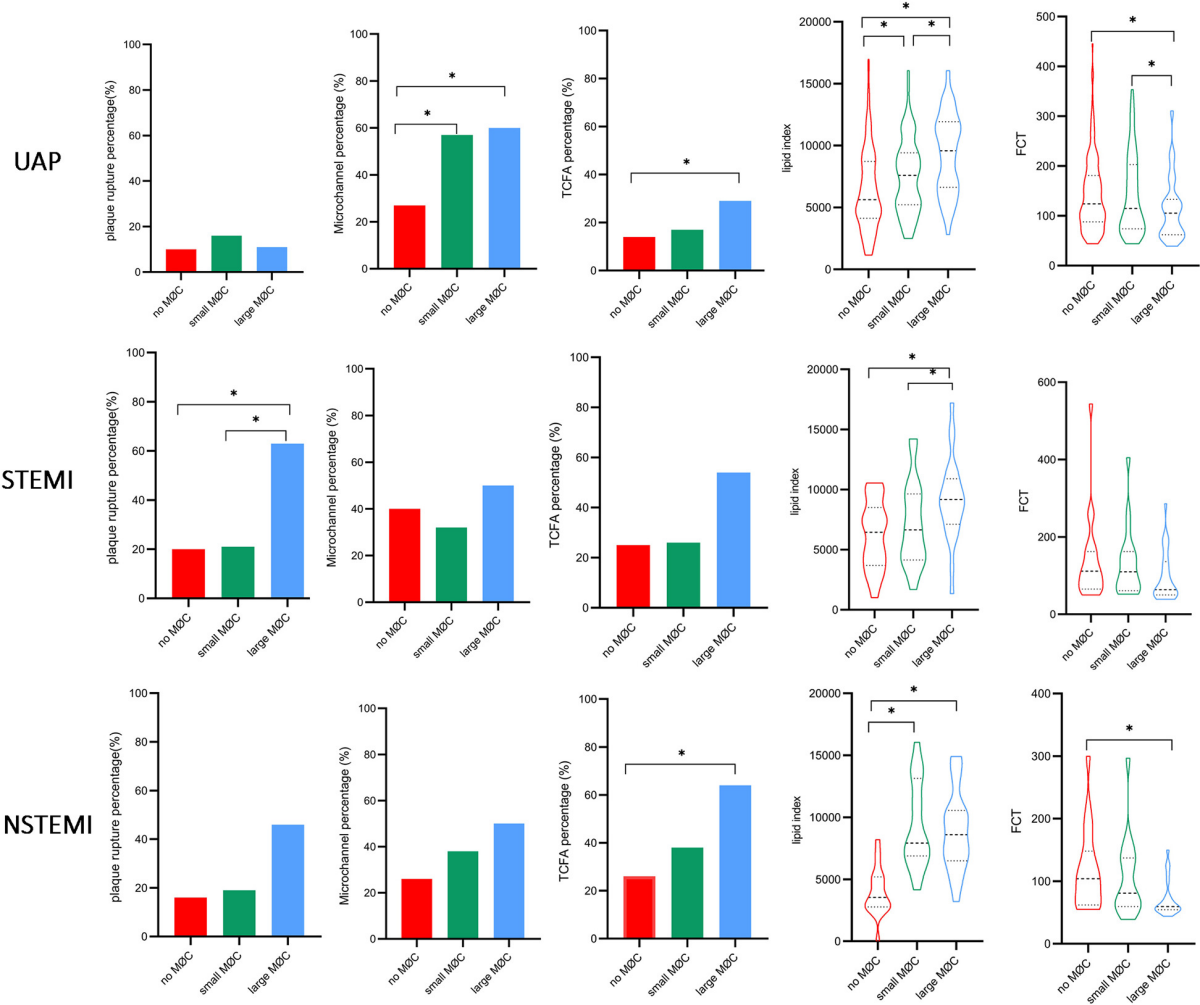


**Figure 8:**
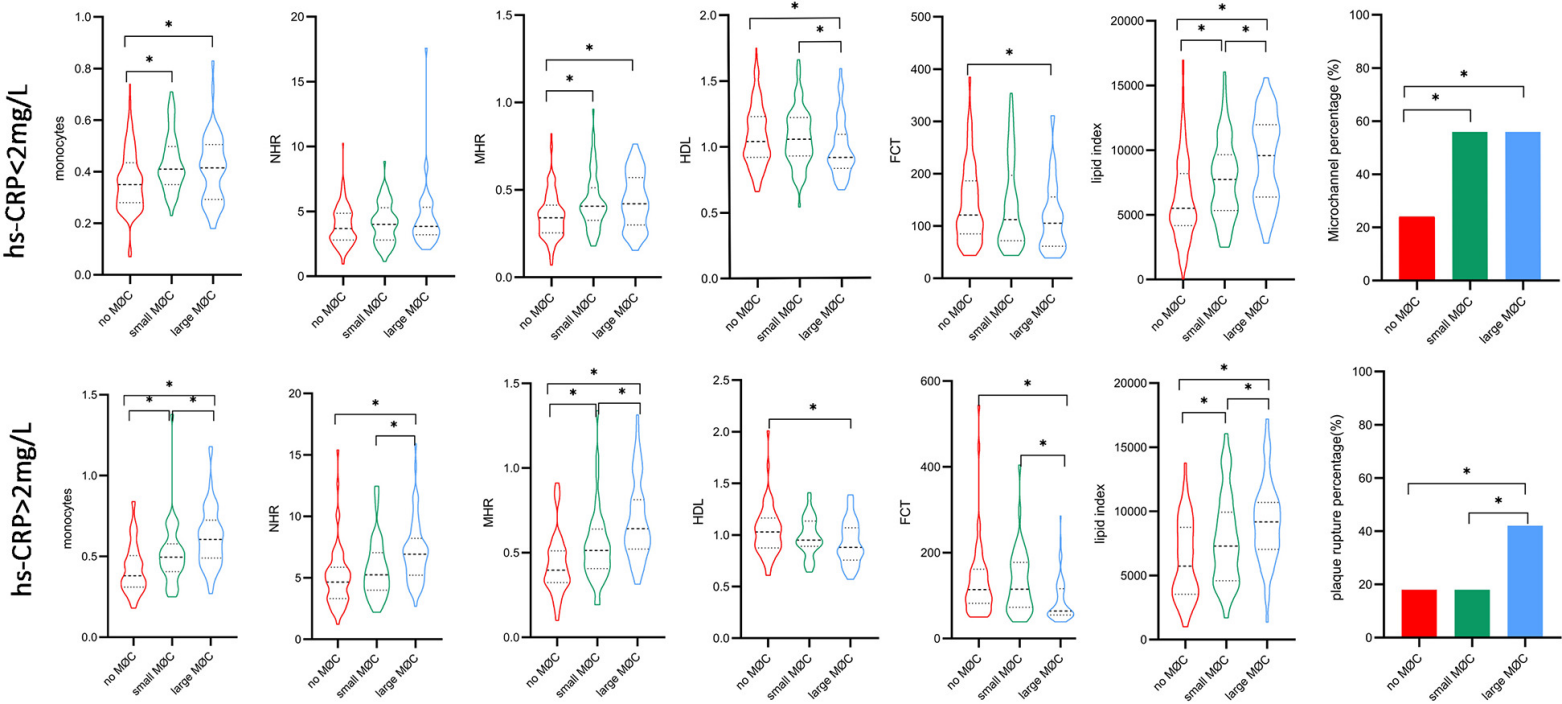
Comparison of inflammation biomarkers and plaque characteristics between macrophage cluster groups in patients with different hs-CRP levels. *indicated *p* < 0.05.

Subgroup analysis according to hs-CRP level showed significant differences in monocytes, MHR, and FCT between the large macrophage cluster group and the no macrophage cluster group in patients with hs-CRP < 2 mg/L, and the differences were more pronounced among the three macrophage cluster groups in patients with hs-CRP ≥ 2 mg/L (Figure 8). A significant relationship existed between lipid index and the circumferential extension of macrophage clusters, irrespective of CRP level. The prevalence of microchannels and layered plaque was higher in the large macrophage cluster group compared to the no macrophage cluster group in patients with hs-CRP < 2 mg/L, and similar trends in plaque rupture and TCFA were observed in patients with hs-CRP ≥ 2 mg/L. Other vulnerable plaque characteristics (microchannels, layered plaque, and cholesterol crystals) did not differ between the three macrophage cluster groups in patients with hs-CRP ≥ 2 mg/L. Interestingly, in patients with hs-CRP < 2 mg/L, WBC, neutrophils, and NHR did not differ significantly between the three macrophage cluster groups. In contrast, higher levels of these biomarkers were observed in the large macrophage cluster group compared with the no macrophage cluster group in patients with hs-CRP ≥ 2 mg/L (Supplement Table S4).

## Discussion

The main findings of this present study of macrophage accumulation were as follows: (1) The assessments of the presence and amount of macrophage clusters were feasible in the quantification of local macrophage accumulation; (2) MHR, lipid index, and microchannel were independently associated with macrophage clusters. The combined three indicators exhibit better predictive ability for the presence of macrophage clusters; (3) The presence and amount of local macrophage clusters were independently associated with MHR and lipid plaque burden, irrespective of the grades of inflammatory background (Graphical Abstract).

Macrophages aggregated in the vulnerable atherosclerotic plaques. Compared with scattered macrophage infiltration, macrophage clusters may better reflect the level of local inflammation. Francesco Prati et al. (8) found macrophage clusters in 577 out of 1,003 patients with coronary heart disease (58%), and the median arc of the macrophage cluster was 67° in the non-culprit left anterior descending; the different culprit lesions may account for the discrepancy in the cutoffs. The macrophages were derived from circulating monocytes and proliferated locally in the atherosclerosis plaque. Though tissue-resident macrophages mostly maintained and replenished themselves by proliferation independently of circulating monocytes in steady-state conditions (19), resident macrophage proliferation was insufficient to sustain generations of macrophages, and new circulating monocytes were recruited during plaque progression (20). Besides, genetic fate mapping studies also reported that the resident macrophages were replaced by an influx of bone marrow-derived monocytes in acute myocardial infarction in mice (21). Previous studies have shown that the number of circulating monocytes was associated with the number of macrophages in the plaque in hypercholesterolemic mice (22). This current study demonstrated, for the first time, that local macrophage clusters were positively correlated with circulating monocytes in humans. Though previous studies reported the relationship between macrophage infiltration and hs-CRP (10), hs-CRP is not an independent factor for macrophage clusters in multivariate analysis in this current study. This discrepancy might be attributed to the limitation of hs-CRP as a marker of local inflammation. The relationship between systemic and local inflammatory responses may be further assessed by non-invasive imaging (23, 24).

Abundant evidence supported that HDL and its associated apolipoproteins, enzymes, and lipids had an essential impact on the modulation of immune cell activation and function (25). HDL and its associated apoA-I and paraoxonase-1 prevented monocyte inflammatory response and macrophage proinflammatory phenotype through suppressing reactive oxygen species production and inflammatory signaling (lipopolysaccharide, toll-like receptor, and nuclear factor kappa B), as well as transportation of free cholesterol from macrophage foam cells to the liver (26). A recent study demonstrated that patients with high levels of coronary inflammation [evaluated by peri-coronary adipose tissue (PCAT) attenuation] had lower levels of HDL (27). In agreement with previous studies, our results showed a negative relationship between HDL and macrophage accumulation. Furthermore, this current study revealed that combining the detrimental effect of monocytes and the protective effect of HDL promised MHR a more valuable predictor of plaque inflammation, irrespective of clinical presentation and inflammatory background.

Deposition of lipids in the arterial intima triggered an inflammatory response characterized by monocyte recruitment and macrophage differentiation. Our study demonstrated that the higher lipid plaque burden was related to larger local macrophage clusters, regardless of the inflammation level. This is consistent with a previous report that higher noncalcified plaque volume was associated with higher macrophage accumulation and plaque vulnerability (28). Similarly, a positive correlation was observed between PTCA attenuation and noncalcified plaque volume and low-density non-calcified plaque volume burden (29, 30). Based on these findings, we hypothesized that a higher lipid plaque burden provided more vulnerable sites for monocyte recruitment, adherence, and differentiation. Moreover, our study demonstrated that microchannels were independently associated with macrophage clusters. The combined indicator (MHR, lipid index, and microchannels) exhibited better predictive ability for the presence of macrophage clusters than each individual component, indicating the intricate association between local coronary plaque inflammation, systemic inflammation, local plaque structure, and plaque burden. The lower macrophage cluster arc in the low MHR + low lipid index group suggested that MHR and lipid burden may synergistically affect macrophage infiltration within local plaques.

Superficial macrophage accumulation could shadow the underlying tissue and appear as a TCFA in OTC imaging (31). Several OCT features supported luminal macrophage accumulation instead of TCFA: well-delineated, sharp radial borders in several adjacent frames and visualized underlying tissue between the multiple shadows (32). However, the OCT imaging could not provide a reliable analysis of radial borders of superficial macrophage clusters in lipid-rich plaque with a large necrotic core or massive thrombus so that the macrophage clusters may be underestimated in these lesions. The underestimate for the circumferential extension of large macrophage clusters may account for the lack of significant differences between the high MHR + high lipid index group, the high MHR + low lipid index group, and the low MHR + high lipid index group.

### Study limitation

First, the low accuracy in the culprit lesion with a large necrotic core or massive thrombus may result in an underestimate of macrophage clusters, so a plaque-based comparison of OCT findings was not performed. We did not compare macrophage clusters between culprit sites and remote sites for the same reason, though the previous study had reported that macrophage density at remote lesions correlated significantly with that of culprit lesions (33). Meanwhile, this retrospective study analyzed the OCT imaging only in the culprit vessel but not in the three vessels. Second, macrophage clusters were not identified and compared with matching histological findings and computed macrophage density (NSD). Third, selection bias cannot be excluded due to the relatively limited number of STEMI and NSTEMI patients in this retrospective, single-center study. Fourth, our study did not use other proinflammatory biomarkers and lipid profiles, such as interleukins (IL-6, IL-1β), reactive oxygen species, matrix metalloproteinase, lipoprotein(a), and apolipoprotein B. Fifth, OCT image analysis was not performed in an independent core laboratory, and long-term follow-up of our patients was unavailable at this time. A further prospective study is needed to clarify whether macrophage clusters provide additive predictive value beyond well-established OCT-based predictors.

## Conclusions

In conclusion, this study provides the first *in vivo* data exploring the application of macrophage clusters in the quantitative analysis of coronary plaque inflammation. Our study demonstrated that MHR, lipid index, and microchannel were independently associated with macrophage clusters. Moreover, the combined three indicators exhibit better predictive ability for macrophage clusters. Large macrophage clusters were independently associated with high MHR and high lipid plaque burden, irrespective of inflammatory background. Macrophage clusters might therefore emerge as a valuable quantification index of local macrophage accumulation. However, further larger prospective studies are needed to assess the prognostic value of macrophage clusters.

## Data Availability

The original contributions presented in the study are included in the article/Supplementary Material, further inquiries can be directed to the corresponding authors.
