# Supplementary material for: The relationships between inflammatory biomarkers, plaque characteristics, and macrophage clusters in coronary plaque: a quantitative assessment of macrophages based on optical coherence tomography
Source: Front Cardiovasc Med. 2025 Jun 25;12:1625239. doi: 10.3389/fcvm.2025.1625239 (PMC12238026; doi:10.3389/fcvm.2025.1625239)
Supplement: Supplementary file 1 [file Supplementaryfile1.docx]

Detailed information of the OCT examination:

Definition of OCT-based parameters[1]: Lipid plaque was defined as a signal-poor region diffusely bordered by overlying signal-rich bands with a lipid arc of >180°; Thin cap fibroatheromas (TCFA) were described as a lipid-rich plaque with the thinnest fibrous cap ≤65 μm and lipid arc of >90°.Red thrombi (erythrocyte-rich): highly backscattering and high attenuation (resembling blood); white thrombi (platelet-rich): less backscattering, homogeneous, and lower attenuation; Layered plaque is defined as plaque with one or more layers of different optical densities and a clear demarcation from underlying components; Calcified plaques are signal-poor regions with sharply delineated borders and limited shadowing; The calcium index was defined as the product of the mean calcium arc and the total calcium length. A macrophage cluster was defined as a large accumulation of confluent bright spots on plaque surfaces, accompanied by high attenuation. Microchannels in the intima can appear in OCT images as signal-poor voids that are sharply delineated and can usually be followed in multiple contiguous frames. Cholesterol crystals are defined as thin, linear regions of high intensity, sharp-bordered, and sometimes stacked structures on OCT images. High attenuation is rare in cholesterol crystals but common in macrophage clusters; this may be an important distinguishing feature between cholesterol crystals and macrophage clusters. Plaque rupture is characterized by the presence of a fibrous cap discontinuity with a cavity formation within the plaque. Definite plaque erosion is characterized by the presence of attached thrombus overlying an intact and visualized plaque. Probable plaque erosion is characterized by luminal surface irregularity at the culprit lesion in the absence of thrombus. References

[1] Araki M, Park SJ, Dauerman HL, Uemura S, Kim JS, Di Mario C, et al. Optical coherence tomography in coronary atherosclerosis assessment and intervention. Nat Rev Cardiol. 2022;19:684-703.

Supplement table 1 Comparison between four groups stratified by the median value of MHR and lipid index

|  | low MHR+ low lipid index (n=55) | high MHR+ low lipid index (n=59) | low MHR+ high lipid index (n=54) | high MHR+ high lipid index (n=50) | P |
| --- | --- | --- | --- | --- | --- |
| Plaque rupture | 8 (15%) | 7 (12%) | 16 (30%) | 20 (40%) | 0.001 |
| Layered plaque | 22 (40%) | 19 (32%) | 24 (44%) | 17 (34%) | 0.528 |
| Cholesterol crystal | 10 (18%) | 15 (25%) | 19 (35%) | 16 (32%) | 0.201 |
| FCT (μm) | 135.4±78.8 | 131.0±85.8 | 115.8±65.6 | 91.9±45.2 | 0.009 |
| Microchannel | 27 (49%) | 25 (42%) | 32 (59%) | 31 (62%) | 0.140 |

MHR = monocyte/HDL ratio; FCT = fibrous cap thickness;

Supplement Table 2 The comparison of MHR, lipid index, and microchannel between groups based on the circumferential extension tertiles.

|  | No MØC  (<50°, n=186) | Small MØC  (50-64°, n=74) | Intermediate MØC (65-86°, n=77) | Large MØC  (>86°, n=67) | P |
| --- | --- | --- | --- | --- | --- |
| MHR | 0.374±0.150 | 0.463±0.186 | 0.511±0.186 | 0.606±0.273 | <0.001 |
| Lipid index | 6170±3152 | 7469±3180 | 8915±3340 | 9012±3241 | <0.001 |
| Microchannel (%) | 52 (28%) | 35 (47%) | 43 (56%) | 37 (55%) | <0.001 |

MHR = monocyte/HDL ratio;

Supplement Table 3 Comparison of systemic inflammation levels between UAP, NSTEMI, and STEMI.

|  | UAP  (n=284) | NSTEMI (n=57) | STEMI  (n=63) | P _UAP vs NSTEMI_ | P _NSTEMI VS STEMI_ |
| --- | --- | --- | --- | --- | --- |
| WBC | 6.53±1.80 | 7.94±2.24 | 9.21±2.4 | <0.001 | <0.001 |
| neutrophils | 4.11±1.53 | 5.50±2.20 | 6.67±2.13 | <0.001 | <0.001 |
| monocytes | 0.398±0.124 | 0.514±0.188 | 0.584±0.205 | <0.001 | 0.011 |
| NHR | 4.17±1.87 | 5.69±2.42 | 6.93±2.69 | <0.001 | 0.001 |
| MHR | 0.403±0.158 | 0.540±0.230 | 0.615±0.267 | <0.001 | 0.030 |
| hs-CRP | 2.07±3.21 | 5.79±6.25 | 7.93±7.23 | <0.001 | 0.010 |

UAP = unstable angina pectoris; NSTEMI = non-ST-segment elevation myocardial infarction; STEMI = ST-segment elevation myocardial infarction; WBC = white blood cells; NHR = neutrophil/HDL ratio; MHR = monocyte/HDL ratio; hs-CRP = high-sensitivity C-reactive protein.

Supplement table 4 Comparison of systemic inflammation levels between groups based on the hs-CRP level.

|  | hs-CRP< 2mg/L (n=247) | | |  | hs-CRP≥ 2mg/L (n=157) | | |  |
| --- | --- | --- | --- | --- | --- | --- | --- | --- |
|  | No MØC  (n=129) | Small MØC  (n=70) | Large MØC  (n=48) | P | No MØC  (n=57) | Small MØC  (n=40) | Large MØC  (n=60) | P |
| WBC | 6.26±1.55 | 6.66±1.47 | 6.65±2.22± | 0.185 | 7.38±2.44 | 8.14±2.22 | 9.14±2.39 | <0.001 |
| neutrophils | 4.00±1.32 | 4.17±1.38 | 4.23±2.01 | 0.588 | 5.15±2.46 | 5.46±2.13 | 6.34±2.06 | 0.014 |
| NHR | 3.88±1.47 | 4.09±1.55 | 4.44±2.43 | 0.148 | 5.09±2.52 | 5.73±2.42 | 7.12±2.56 | <0.001 |
| monocytes | 0.364±0.116 | 0.436±0.110 | 0.412±0.133 | <0.001 | 0.424±0.153 | 0.515±0.194 | 0.616±0.186 | <0.001 |
| MHR | 0.351±0.130 | 0.430±0.160 | 0.436±0.160 | <0.001 | 0.429±0.178 | 0.542±0.222 | 0.692±0.236 | <0.001 |
| Lipid index | 6177±3215 | 7705±3100 | 9296±3374 | <0.001 | 6155±3033 | 7778±3505 | 9106±3175 | <0.001 |
| Layered plaque | 26 (20%) | 28 (40%) | 16 (33%) | 0.009 | 22 (39%) | 14 (35%) | 24 (40%) | 0.878 |
| Cholesterol crystal | 21 (16%) | 16 (23%) | 15 (31%) | 0.086 | 10 (18%) | 12 (30%) | 17 (28%) | 0.274 |
| Microchannel | 31 (24%) | 39 (56%) | 27 (56%) | <0.001 | 21 (37%) | 16 (40%) | 33 (55%) | 0.113 |
| Plaque rupture | 11 (9%) | 12 (17%) | 7 (15%) | 0.175 | 10 (18%) | 7 (18%) | 25 (42%) | 0.004 |
| Plaque erosion | 2 (2%) | 3 (4%) | 0 (0%) | 0.230 | 5 (9%) | 1 (3%) | 1 (2%) | 0.139 |
| TCFA | 20 (16%) | 15 (21%) | 15 (31%) | 0.065 | 11 (19%) | 9 (23%) | 30 (50%) | 0.001 |

Values are mean±SD, n (%), or median (25th–75th percentile).

hs-CRP = high-sensitivity C-reactive protein. MØC = macrophage cluster; WBC = white blood cells; NHR = neutrophil/HDL ratio; MHR = monocyte/HDL ratio; TCFA = Thin cap fibroatheromas.
